# Recent Advances in Host-Directed Therapies for Tuberculosis and Malaria

**DOI:** 10.3389/fcimb.2022.905278

**Published:** 2022-05-20

**Authors:** Kely C. Matteucci, André A. S. Correa, Diego L. Costa

**Affiliations:** ^1^ Plataforma de Medicina Translacional Fundação Oswaldo Cruz/Faculdade de Medicina de Ribeirão Preto, Universidade de São Paulo, Ribeirão Preto, Brazil; ^2^ Departamento de Bioquímica e Imunologia, Faculdade de Medicina de Ribeirão Preto, Universidade de São Paulo, Ribeirão Preto, Brazil; ^3^ Programa de Pós-Graduação em Imunologia Básica e Aplicada, Faculdade de Medicina de Ribeirão Preto, Universidade de São Paulo, Ribeirão Preto, Brazil

**Keywords:** tuberculosis, malaria, host-directed therapy, immunity, intracellular development, pathogenesis, cell invasion

## Abstract

Tuberculosis (TB), caused by the bacterium *Mycobacterium tuberculosis*, and malaria, caused by parasites from the *Plasmodium* genus, are two of the major causes of death due to infectious diseases in the world. Both diseases are treatable with drugs that have microbicidal properties against each of the etiologic agents. However, problems related to treatment compliance by patients and emergence of drug resistant microorganisms have been a major problem for combating TB and malaria. This factor is further complicated by the absence of highly effective vaccines that can prevent the infection with either *M. tuberculosis* or *Plasmodium*. However, certain host biological processes have been found to play a role in the promotion of infection or in the pathogenesis of each disease. These processes can be targeted by host-directed therapies (HDTs), which can be administered in conjunction with the standard drug treatments for each pathogen, aiming to accelerate their elimination or to minimize detrimental side effects resulting from exacerbated inflammation. In this review we discuss potential new targets for the development of HDTs revealed by recent advances in the knowledge of host-pathogen interaction biology, and present an overview of strategies that have been tested *in vivo*, either in experimental models or in patients.

## Introduction

Tuberculosis (TB) and malaria have been a huge public health problem for humanity for centuries and still presently account for two of the deadliest infectious diseases of mankind (WHO, 2019; WHO, 2021). TB is caused by the infection with the bacterium *Mycobacterium tuberculosis*, while malaria is caused by the infection with protozoan parasites from the genus *Plasmodium* (*P. falciparum*, *P. vivax*, *P. ovale*, *P. malariae* and *P. knowlesi*). The most recent data from the World Health Organization show that in 2020, approximately 10 million new cases of active TB were recorded and nearly 1.5 million people died from the disease (WHO, 2021). For malaria, data from 2019 indicate that an estimated 229 million people contracted the disease, while nearly 409,000 died from it during that year (WHO, 2019). Moreover, the advent of COVID-19 pandemics by the end of 2019 and beginning of 2020 represented a massive setback for the programs of prevention and treatment of these illnesses. Due to the need for repurposing budget, human resources, health facilities and diagnostic machinery for fighting the COVID-19 pandemics, estimates indicate that there was a sharp decrease in TB diagnostic and treatment coverage, while for malaria, these parameters have stalled after a long series of continuous improvements (The Global Fund Results Report, 2021).

The only vaccine available for TB prevention, BCG – discovered nearly a century ago, offers poor protection against adult pulmonary TB (Soysal et al., 2005; Tuberculosis Research, 2013). Several research groups have tried to develop new vaccines for TB and there are various candidates undergoing clinical trials, however, no candidate has been licensed since BCG introduction (WHO, 2021). Only recently a vaccine for malaria was approved for human use, however, there is not enough data to draw strong conclusions regarding its protection effectiveness against infection with different *Plasmodium* species. The results of a phase III trial conducted with more than 15 thousand children in Africa demonstrated that it induces close to 50% protection during the first year of administration, but over a follow up period of 4 years, the protection rate drops to 28 and 36% respectively, depending on having received or not a booster dose (Rts, 2015). Nevertheless, there are high hopes that it is going to be an important tool to help controlling malaria, since due to the huge number of annual cases, these rates of protection would result in a marked reduction in disease prevalence and mortality.

Pharmacological treatment is available for both TB and malaria, however, problems related to the effectiveness of these treatments have been on the rise in recent years (WHO, 2019; WHO, 2021). The conventional therapy for TB consists of six to nine months of continuous daily antibiotic administration (Rifampicin + Isoniazid + Pyrazinamide + Ethambutol for 2 months followed by four to seven months of Rifampicin + Isoniazid administration) (WHO, 2010). Due to this lengthy therapeutic regimen composed by several different drugs, the occurrence of side effects is common and many patients abandon treatment after initial improvement of symptoms. This promotes reactivation of disease with selection of drug resistant bacilli. Multidrug resistant TB is a major problem for disease control and requires the use of more toxic drugs, which need to be administered for longer periods and have a reduced success rate (WHO, 2021). The pharmacological treatment of malaria employs different drugs that may be administered alone or in combination depending on the parasite species, host age as well as disease severity and chronicity. Some of the most common antimalarial drugs in use are: quinine, chloroquine, amodiaquine, mefloquine, primaquine, artemisinin, artesunate, artemether and sulfadoxine/pyrimethamine. Despite the wide range of antimalarials, the development of drug resistance in *Plasmodium* is common, particularly in *P. falciparum* and *P. vivax* species, and poses a major challenge for malaria control (Tse et al., 2019).

Much effort and investment in the research of novel drugs for both TB and malaria has been continuously employed throughout the last decades. However, for TB, many of the drugs developed over the years have serious host toxicity issues, while for malaria, the rapid development of parasites resistant to the new drugs has always been a major problem (Ramachandran and Swaminathan, 2015; Belete, 2020; Yang et al., 2022). Nevertheless, in both TB and malaria, the host immune and inflammatory responses as well as basic cellular physiologic mechanisms, play critical roles in disease establishment and progression. Therefore, the host biological processes that favor pathogen fitness and/or pathogenesis can be targeted for the development of new therapies. Such Host-Directed Therapies (HDTs) usually aim at optimizing host immune responses and improving microbicidal activity of phagocytes and infected cells, restraining pathogen development within other non-hematopoietic host cells, or at dampening exacerbated inflammation that may cause damage to host tissues (Zumla et al., 2016). The major advantage of these treatments lies on the fact that by targeting the host and not the pathogen itself, they are not expected to promote the selection of drug resistant organisms. Moreover, pathogens that are resistant to conventional drugs should be equally susceptible to the HDTs as drug susceptible microorganisms are. Therefore, HDTs are promising strategies that can help fighting both TB and malaria.

In this review article we discuss potential targets for HDTs for both of these devastating diseases based on current knowledge regarding host-pathogen interactions. We also review results from articles in which HDT approaches were tested in animal models as well as clinical trials.

## Host-Directed Therapies for Tuberculosis

The aetiologic agent of TB, *M. tuberculosis* (Mtb) is transmitted mainly through the air, after a healthy individual inhales bacteria contained in droplets of sputum expelled by an infected person, and, therefore, the lungs are the primary organs affected by the disease. Once in the alveoli, Mtb bacilli will be internalized by phagocytes, mainly alveolar macrophages and dendritic cells (DCs), and the efficient induction of microbicidal effector mechanisms in those cells is able to restrict the infection in many cases (Ernst, 2012). At the same time, dendritic cells that have phagocytosed bacteria migrate to the mediastinal draining lymph nodes, where they present Mtb antigens to T cells. The production of IL-12 by the DCs during antigen presentation promotes the activation and differentiation of naïve CD4^+^ T lymphocytes into Th1 cells, which will then secrete IFN-γ and TNF upon recognition of Mtb antigens in MHC class II molecules present on the surface of infected macrophages in the lungs (Cooper, 2009). These cytokines play a major role in the activation of infected cells and induce the production of reactive oxygen and nitrogen species (ROS and RNS, respectively), resulting in killing of the bacteria (Shiloh and Nathan, 2000). In addition, DCs also present Mtb antigens to naïve CD8^+^ T cells, resulting in their activation, differentiation into cytotoxic CD8^+^ T lymphocytes and further proliferation. After migrating to the infection sites, Mtb-specific CD8^+^ cytotoxic T lymphocytes recognize Mtb antigens in MHC class I molecules present on the surface of infected cells and release granzymes, perforin and granulysin, which will kill the Mtb-infected phagocytes (Lin and Flynn, 2015). This cooperative action of adaptive and innate immune responses can eliminate those bacteria that have escaped the initial host innate effector mechanisms in most of the cases.

However, Mtb have evolved numerous strategies to subvert host immune responses and, in doing so, is able to survive and replicate in infected cells even in face of immune pressure (Zhai et al., 2019). When the bacteria escape host innate and adaptive immune responses, the development of a chronic infection occurs, which can present as asymptomatic (latent) or active. In those scenarios, while the dynamic immune response to Mtb evolves, the classical hallmark of TB disease also develops, which is the establishment of pulmonary granulomas (Pagan and Ramakrishnan, 2014). In the TB granulomas, infected phagocytes (such as monocyte-derived macrophages, foamy macrophages, multinucleated giant cells and neutrophils) are confined to a central core, which is surrounded by macrophages that develop an epithelioid phenotype (epithelioid macrophages) and a secondary surrounding layer composed mainly of lymphocytic cells (mainly CD4^+^ T, CD8^+^ T and B lymphocytes). In the central core, cells that do not contain bacterial replication efficiently may undergo necrotic cell death, which ultimately results in the development tissue necrosis (caseous necrosis). An outer layer of fibroblasts also develops along with inflammation chronicity, which results in deposition of collagen fibers around the granuloma walls (Ramakrishnan, 2012; Guirado and Schlesinger, 2013).

When stable granulomas are formed, bacterial spread is contained, while the immune pressure provided by the cells that form the structure induce Mtb bacilli to undergo latency, a state in which bacteria remain viable, but display extremely low metabolic and replication rates (Salgame et al., 2015). Patients with latent TB do not present disease symptoms nor transmit it, although they have 5–10% risk of developing active TB during their lifetime (O'Garra et al., 2013). Additionally, estimates indicate that around 1.7 billion people have latent TB in the world (Houben and Dodd, 2016). It is important to mention that in around 95% of the cases, host immune and inflammatory responses control the infection, resulting in bacterial elimination or efficient containment of latent bacilli within granulomas. However, in the remaining percentage of infected people, all of those host mechanisms fail to restrict Mtb replication and active TB develops (Sutherland et al., 1982; Sloot et al., 2014; Erkens et al., 2016). Active pulmonary TB is a heterogenous disease, which may manifest as single or multiple lesions, affecting one or both lungs, and it is also possible to find cavitary lesions along with developing granulomas containing actively dividing bacteria side by side with stable granulomas hosting latent bacilli, all within the same patient (Cadena et al., 2017).

As mentioned previously, the production of TNF and IL1-β by innate immune cells, the development of effective Th1 responses characterized by IFN-γ and TNF production, as well as the formation of stable granulomas, have all been shown to be critical for the host to control bacterial replication and dissemination in TB (Cooper et al., 1993; Flynn et al., 1995; Algood et al., 2005; Mayer-Barber et al., 2010). However, in the setting of active disease, the chronic production of these same otherwise protective cytokines in excess may result in necrotic host cell death, tissue destruction and promotion of bacterial dissemination (Bekker et al., 2000; Algood et al., 2005; Mishra et al., 2013; Sakai et al., 2016), and depending on their structure composition, granulomas can restrict the access of antimycobacterial drugs to the infected cells contained in their central core (Dartois, 2014).

**Figure 1:**
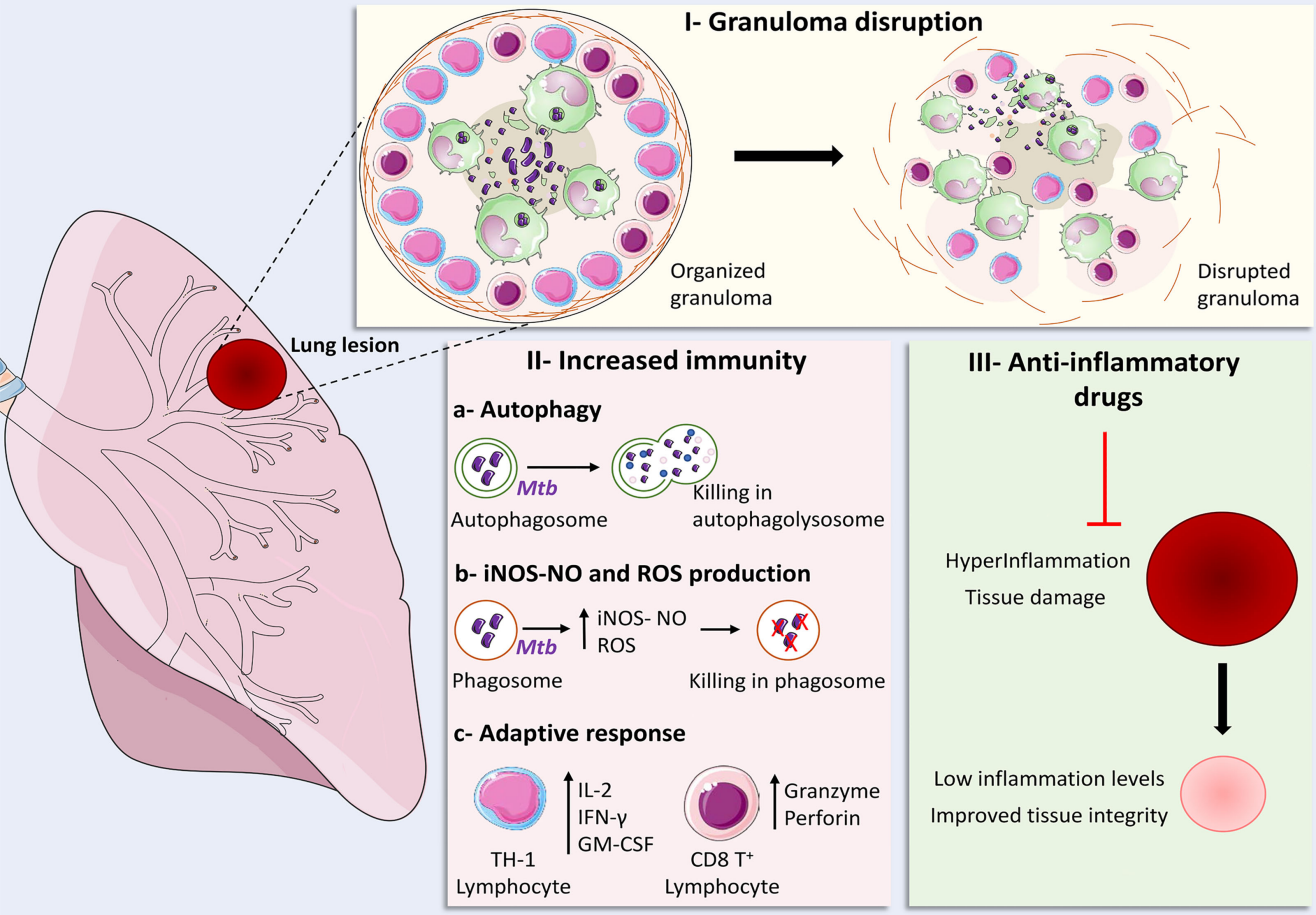
Main strategies for the development of Host-Directed Therapies for tuberculosis. I – Granuloma disruption: strategies focused at granuloma disruption employed in conjunction with antibiotic therapy aim to loosen granuloma structure in order to promote improved drug penetration and lymphocyte migration to regions where infected cells are located; II – Increased immunity: **(A)** enhancement of autophagy-mediated bacterial elimination, **(B)** ROS and iNOS-NO-dependent bacillary killing, as well as **(C)** increased Th1 responses and cytotoxic CD8^+^ T cell effector activity can be achieved though infusion of cytokines, administration of repurposed drugs and nutritional supplementation; III – Anti-inflammatory drugs: The use of several families of anti-inflammatory drugs were tested in experimental models and clinical trials in order to restrict the damage inflicted to host tissues by exaggerated inflammatory responses, which resulted in decreased or optimized production of pro-inflammatory mediators, improving tissue integrity and immune responses against the bacteria. Some elements in this figure use pictures from Servier Medical Art (https://smart.servier.com) licensed under a Creative Commons Attribution 3.0 Unported License (https://creativecommons.org/licenses/by/3.0/).

Considering all of these different possible scenarios, the main goals of HDT strategies to TB are focused at three major objectives: a) improvement of immune responsiveness and microbicidal activity of infected cells, b) mitigation of exacerbated pulmonary inflammation and c) modulation of granuloma structure (**Figure 1**). At the following sections we have reviewed some of those strategies that were tested *in vivo*, either in experimental models or clinically.

### Enhancement of Host Immunity and Bactericidal Activity

Following inhalation of Mtb bacilli by a healthy individual, the pathogens first encounter host pulmonary innate immune cells, which recognize bacterial molecules through pattern recognition receptors (PRRs), such as Toll-like receptors (TLRs), Nod-like receptors (NLRs) and C-type lectin receptors (CLRs). These receptors trigger the initiation of several innate immune effector functions that can restrict bacterial replication in host cells (Killick et al., 2013; Lerner et al., 2015; Sia et al., 2015). Khan et al. found that Mtb-infected mice treated with agonists of CD40 and TLR-4 (C40.T4) in conjunction with isoniazid display enhanced bacterial clearance in lungs compared to animals treated with each regimen alone. The beneficial effects were associated with increased production of IL-12, TNF and IL-6 by dendritic cells as well as IFN-γ by CD4^+^ and CD8^+^ T cells and IL-17 by CD4^+^ T cells (Khan et al., 2016).

The supplementation with cytokines known to play important roles in the host resistance to *M. tuberculosis* infection have also been tested as HDTs for TB in different settings. IL-12-induced differentiation of IFN-γ producing Th1 lymphocytes, as mentioned previously, is critical for the host resistance to TB (Cooper et al., 2007). In a clinical case study, it was demonstrated that IL-12 administration to a patient with disseminated TB who was unresponsive to conventional treatment restored antibiotic efficacy in controlling bacterial replication (Greinert et al., 2001). Also, administration of IFN-γ *via* aerosol to patients with MDR-TB (500 μg – 3x/week for 1 month) resulted in negative sputum smears and improvement in radiological scores of pulmonary lesions (Condos et al., 1997). However, a separate article reported that administration of IFN-γ through aerosol at higher doses to MDR-TB patients failed to induce sputum smear culture conversion (Koh et al., 2004). In patients infected with drug susceptible Mtb presenting with cavitary lung lesions, the supplementation of recombinant IFN-γ 1b by aerosol as an adjunct to conventional antibiotic therapy resulted in reduction of pro-inflammatory cytokine production and enhancement of CD4^+^ lymphocyte responsiveness to PPD, along with higher rates of sputum conversion, compared to antibiotic treatment alone (Dawson et al., 2009).

IL-2 also plays an important role in the development of CD4^+^ and CD8^+^ T cell-mediated adaptative immunity to Mtb infection (Suter-Riniker et al., 2011). Recombinant IL-2 supplementation in conjunction with drug treatment was shown to enhance cellular immunity, increase the rates of sputum conversion and improve radiological scores in patients with MDR-TB (Johnson et al., 1997; Shen et al., 2015; Tan et al., 2017).

GM-CSF is another cytokine that was demonstrated to play an important role in host resistance to TB (Rothchild et al., 2017; Bryson et al., 2019). Utilizing an experimental model of TB, Zhang et al. found that GM-CSF treatment of drug-susceptible Mtb-infected mice resulted in reduction of bacterial loads in lungs and spleens, while in drug resistant Mtb infected animals, the combination of GM-CSF and IL-2 was even more effective than each cytokine alone in promoting increased resistance to infection (Zhang et al., 2012). In patients with pulmonary TB, a trend to faster sputum conversion was observed following treatment with GM-CSF in conjunction with standard therapy compared to antibiotic treatment alone, although no significant difference was found (Pedral-Sampaio et al., 2003).

Several classes of drugs utilized in the treatment of a number of conditions have been repurposed for the use as HDTs for TB and found to enhance the capacity of host infected cells in controlling bacterial replication. Following phagocytosis of Mtb bacilli, phagosome acidification and maturation followed by fusion with lysosomes can eliminate intracellular Mtb (Pauwels et al., 2017; Queval et al., 2017). The production of ROS *via* NADPH oxidase 2 (NOX2) and RNS utilizing iNOS-derived NO, play an important role in the killing intraphagosomal Mtb (Bogdan et al., 2000; Nathan, 2003). Autophagy can also function as an innate defense mechanism against Mtb infection by promoting elimination of bacilli contained both in phagosomes or in the cytoplasm (Jo, 2013; Kim et al., 2019).

Metformin, for example, is a drug commonly used in the treatment of type II diabetes mellitus (DM) (Pernicova and Korbonits, 2014), which has been tested as an HDT for TB in animal models and also clinical trials. Mice infected with Mtb and treated with isoniazid plus metformin or ethambutol plus metformin displayed reduced bacterial loads and lung pathology compared to animals administered antibiotics only. This effect was associated with metformin-induced enhanced intracellular killing of Mtb *via* mitochondrial ROS production and higher IFN-γ production by CD8^+^ T cells. The authors also identified that among patients with pulmonary TB-DM comorbidity, those who were treated with metformin displayed reduced frequency of cavitary disease and mortality rates during antibiotic treatment for TB (Singhal et al., 2014). The sputum culture conversion rate was also found to be improved by adjunctive metformin treatment in diabetic patients with cavitary pulmonary TB (Lee et al., 2018). Frenkel et al. found that metformin treatment resulted in decreased bacterial loads in a model of Mtb chronic infection in guinea pigs, although metformin administration adjunctively to antibiotic treatment was not tested in this study (Frenkel et al., 2020). Finally, recent articles in which systematic reviews were performed identified that metformin treatment decreased the risk of TB development in diabetic patients and reduced the mortality of diabetic TB patients (Yu et al., 2019; Zhang and He, 2020).

Statins comprise a class of drugs commonly used in the treatment of hypercholesterolemia, which act as inhibitors of the β-Hydroxy β-methylglutaryl-CoA (HMG-CoA) reductase enzymes (Stancu and Sima, 2001). Statin treatment of Mtb-infected mice resulted in reduction of pulmonary bacterial loads and pathology, which was associated with statin-induced increase in autophagy and phagosome maturation (Parihar et al., 2014). When administered in conjunction with antibiotics, statins accelerated bacterial clearance in mice infected with Mtb (Skerry et al., 2014; Dutta et al., 2016; Dutta et al., 2020). In addition, studies indicate that people who undergo statin treatment present decreased risk of developing active TB (Lee et al., 2015; Lai et al., 2016; Su et al., 2017; Li et al., 2020). As mentioned above, statin treatment promotes major effects on host lipid metabolism, which may also be associated with the improved protection against Mtb-infection. In Mtb-infected macrophages, lipid droplets form in close proximity with bacteria-containing phagosomes. After the phagosomes are surrounded by such lipid bodies, bacteria can use their fatty acid, cholesterol and triglyceride contents for nutrition and survival (Peyron et al., 2008; Roque et al., 2020). In fact, it was demonstrated that activation of the antilipolytic receptor GPR109A in Mtb-infected macrophages promotes lipid body formation, which attenuates the cellular microbicidal effects. The inhibition of GPR109A resulted in improved control of Mtb replication both *in vitro* in THP-1 -infected cells and *in vivo* in Mtb-infected mice (Singh et al., 2012). The activation of Peroxisome Proliferator-Activated Receptor γ (PPARγ) and Testicular Receptor 4 (TR4) by lipids in Mtb-infected foamy macrophages was also demonstrated to upregulate CD36 expression (which favors lipid uptake) and to promote phagolysosome maturation blockade and IL-10 production, all of which favor intracellular bacterial survival in those cells (Mahajan et al., 2012). Although several studies have demonstrated that the formation of lipid bodies can favor Mtb survival in macrophages (Shim et al., 2020), it was recently demonstrated that IFN-γ- and HIF1α-induced lipid body formation in macrophages supports the production of PGE2 and LXB4, which are host protective. Also, in the same study the authors found that Mtb is unable to use IFN-γ-induced lipid bodies as a source of nutrients (Knight et al., 2018).

Carbamazepine is an anticonvulsant drug that has been found to reduce bacterial loads and pathological scores in mice infected with a MDR Mtb strain. The beneficial effect was associated with induction of autophagy in macrophages as well as enhancement of TNF, IL-12 and IL-27 production (Schiebler et al., 2015).

Protein kinase inhibitors comprise a class of drugs that have been developed relatively recently and have been used specially in the treatment of different cancers and some autoimmune diseases (Ferguson and Gray, 2018). The tyrosine kinase inhibitor ibrutinib (Bruton’s tyrosine kinase - BTK - inhibitor) was tested as an HDT for TB in a murine model of infection, and it was found that treatment with this drug resulted in decreased bacterial loads in lungs and mediastinal lymph nodes. The authors found that this effect was associated with induction of autophagy through inhibition of the BTK/Akt/mTOR pathway (Hu et al., 2020). The treatment with gefitinib (Epidermal Growth Factor Receptor – EGFR - inhibitor), another tyrosine kinase inhibitor, was shown to reduce bacterial growth in the lungs of mice infected with Mtb, which was also associated to the induction of autophagy (Stanley et al., 2014). *In vitro* studies later demonstrated that gefitinib treatment of cells results in enhanced lysosomal biogenesis and function along with inhibition of STAT3 signaling (Sogi et al., 2017), which plays a detrimental role in the control of Mtb replication in host cells (Queval et al., 2016; Gao et al., 2018). Accordingly, a study performed by Upadhyay et al. found that the inhibition of STAT3 or IL-10 signaling in Mtb-infected mice by the treatment with ST3-H2A2 (a selective peptide inhibitor of the N-terminal domain of STAT3) or IL10R1-7 (a selective peptide inhibitor for IL-10Ra) respectively, resulted in reduction of pulmonary bacterial loads. The treatment with these inhibitors enhanced the apoptosis-autophagy pathways, as well as iNOS, NADPH oxidase and lysozyme activity in the lungs, while suppressing Arg1 activity (Upadhyay et al., 2018).

Sirtuins comprise a family of proteins that promote signaling pathways involved in the regulation of cell metabolism (Yamamoto et al., 2007; Ye et al., 2017) and that have also been linked to the modulation of inflammation (Mendes et al., 2017). Using a murine model of infection with Mtb, Cheng et al. demonstrated that treatment with a natural (resveratrol) or a synthetic (SRT1720) Sirtuin 1 activator resulted in reduced pulmonary tissue damage and bacterial load, improving the efficacy of antibiotic treatment. These effects were associated with enhanced phagosome maturation and autophagy, resulting in improved control of bacterial replication by macrophage as assessed *in vitro* (Cheng et al., 2017). Inhibition of another member of the Sirtuins family, Sirtuin 2, in conjunction with isoniazid, in mice infected with Mtb, resulted in decreased pulmonary bacterial loads and lung pathology compared to antibiotic treatment alone. This effect was associated with enhanced activation of innate immune cells and T lymphocytes (Bhaskar et al., 2020). Specific sirtuin inhibitors and activators have been developed and are being tested in different conditions but were still not approved for human use. However, there are natural products and drugs that can be repurposed to be used with the goal of targeting sirtuins for TB treatment (Dai et al., 2018; Bharadwaj et al., 2021).

Heme oxygenase-1 (HO-1) is an antioxidant enzyme that catalyzes the degradation of heme into equimolar amounts of biliverdin, carbon monoxide and iron (Costa et al., 2020). The inhibition of HO-1 activity with tin protoporphyrin XI (SnPPIX) promotes increased restriction of bacterial replication by human monocyte derived macrophages (Scharn et al., 2016) as well as murine bone marrow derived macrophages *in vitro* (Costa et al., 2021). SnPPIX treatment of Mtb-infected mice results in reduction of pulmonary bacterial loads and accelerates the elimination of bacilli from lungs when performed in conjunction with antibiotics (Costa et al., 2016). HO-1 inhibition was found to enhance iNOS expression and NO production by Mtb-infected macrophages following activation by IFN-γ produced by T lymphocytes, consequently allowing for a more efficient control of bacterial replication by host cells (Costa et al., 2021). SnPPIX was used experimentally in the past for the treatment of jaundice in children (Drummond and Kappas, 1981), however, there are still no HO-1 inhibitors approved for use in humans.

Supplementation with vitamins, nutritional metabolites and derivatives have been tested as HDTs for TB as well. Vitamin D, in particular, has been widely used in animal models as well as in clinical trials. The first *in vitro* studies reported that vitamin D triggers the production of the antibacterial peptide cathelicidin, which induces the killing of intracellular Mtb in human macrophages and THP-1 cells (Liu et al., 2006; Liu et al., 2007). It was later demonstrated that vitamin D-induced cathelicidin triggers autophagy in human macrophages and subsequent Mtb killing (Yuk et al., 2009). Further studies employing *in vivo* infection in mice, demonstrated that treatment with Vitamin D3 or Calcitriol (active vitamin D3 metabolite) alone does not reduce pulmonary bacterial loads, but calcitriol administration does so when performed in combination with pyrazinamide (Reeme and Robinson, 2016; Zhang et al., 2019).

Systematic reviews and meta-analyses of clinical trials employing vitamin D supplementation in TB patients concluded that it enhances the proportion of sputum smear and culture conversions, but does not shorten the time to conversion (Wu et al., 2018; Jolliffe et al., 2019; Karbalaei et al., 2020). In addition, supplementation of Phenylbutyrate plus Vitamin D to standard TB treatment was also found to be beneficial in comparison to antibiotics alone, resulting in higher rates of sputum conversion (Mily et al., 2015) and reduction of inflammatory responses (Rekha et al., 2018). In a recently published article that reported the results of a large clinical trial in which several HDT strategies for TB were tested, the supplementation with vitamin D2 (ergocalciferol) in conjunction with standard therapy did not promote additional improvement in patients’ respiratory function (Wallis et al., 2021). The treatment with retinoic acid, a metabolite of vitamin A, in experimental TB was found to result in reduced pulmonary bacterial loads and lesions in rats and mice (Yamada et al., 2007; O'Connor et al., 2019). The improvement in control of Mtb infection in rats was associated with an increase in CD4^+^ and CD8^+^ T lymphocytes, NK cells and CD163^+^ macrophages in the infection sites (Yamada et al., 2007).

Inhibition of lactate dehydrogenase, which converts pyruvate into lactate during last step of the glycolytic metabolism of glucose, was shown to reduce the bacterial loads in C57BL/6 Mtb-infected mice and to potentiate the efficacy of Isoniazid therapy in Mtb-infected mice deficient for the iNOS gene (Krishnamoorthy et al., 2020), suggesting that approaches aimed at the glycolytic metabolism of glucose might be host beneficial in TB, particularly in scenarios of highly necrotizing lesions, such as those found in iNOS^-/-^ Mtb-infected mice.

Interference in amino acid metabolism was also tested as an HDT for TB. The enzyme indoleamine 2,3-dioxygenase (IDO) catabolizes the conversion of tryptophan to kynurenine, a process that attenuates the production of IFN-γ by CD4^+^ T cells (Mbongue et al., 2015). Treatment of Mtb-infected macaques with the IDO inhibitor 1-methyl-tryptophan resulted in enhanced penetration of T lymphocytes in granulomas along with increased proliferation and granzyme-expression by these cells, consequently favoring the control of bacterial replication in IDO inhibitor-treated animals (Gautam et al., 2018). Administration of the amino acid L-isoleucine to Mtb-infected mice induced the expression of beta-defensins, which was associated with lower pulmonary bacterial loads and tissue damage (Rivas-Santiago et al., 2011).

The strategies and methods mentioned in this section vary significantly among them (cytokine infusion, repurposed drugs, nutritional supplementation or metabolic reprograming), however, altogether they result in enhancement of host’s infected cells bactericidal mechanisms directly or indirectly by potentiating adaptative T lymphocyte-mediated immunity. Importantly, the vast majority of studies cited in this section evaluate such interventions in pulmonary TB or experimental TB models of lung disease, indicating that approaches aimed at boosting host immunity represent promising HDT strategies particularly in pulmonary TB.

### Immunomodulation and Suppression of Inflammation

As mentioned previously, there are distinct cytokines that are important for the proper control of bacterial replication in TB, however, their exaggerated or chronic production mediates tissue destruction that can favor bacterial dissemination and replication (Bekker et al., 2000; Algood et al., 2005; Mishra et al., 2013; Sakai et al., 2016). IL-1β production, for example, is critical for host protection against Mtb infection (Mayer-Barber et al., 2010), however, its chronic production is involved in the development of granulocytic pulmonary inflammation and tissue damage (Mishra et al., 2013). Inhibition of IL-1 signaling in conjunction with linezolid treatment in Mtb-infected mice reduced lung neutrophilic inflammation and spleen bacterial load in comparison to animals treated with the antibiotic alone, although no difference in pulmonary bacterial load was observed. Additionally, the treatment of Mtb-infected macaques with anakinra (a soluble IL-1 receptor antagonist) plus linezolid reduced lung inflammation in comparison with linezolid treatment alone, but no difference in pulmonary bacterial load was found. Importantly, the bone-marrow toxicity induced by linezolid was milder in macaques that received adjunct anakinra treatment compared to those treated with the antibiotic alone (Winchell et al., 2020).

Non-steroidal anti-inflammatory drugs (NSAIDs) comprise a family of cyclooxygenase (COX) inhibitors, which prevent the production of prostaglandins, prostacyclin and thromboxanes, therefore suppressing inflammation in a number of conditions (Bindu et al., 2020). Some of these drugs, such as aspirin and ibuprofen, have been tested as HDTs for TB. The administration of aspirin at low doses to mice experimentally infected with Mtb either alone or in combination with anti-TB antibiotics resulted in increased mouse survival, which was associated to the downmodulation of inflammatory cytokine production and cell recruitment to lesions (Kroesen et al., 2018). Mtb-infected mice treated with ibuprofen also displayed improved resistance to infection, presenting with lower pulmonary bacillary loads and immunopathology than controls (Vilaplana et al., 2013). In addition, the combined administration of aspririn and ibuprofen with pyrazinamide promoted enhanced treatment efficacy in Mtb-infected mice, resulting in reduced pulmonary bacterial loads in comparison to animals treated with the antibiotic alone (Byrne et al., 2007). In humans with tuberculous meningitis, the administration of aspirin at high dose (1,000 mg) was also found to be protective, since it reduced brain infarction rates and mortality, which was associated with decrease in thromboxane A2 production and upregulation of protectins (Mai et al., 2018).

Zileuton is an anti-inflammatory drug that inhibits the activity of the 5-lipoxygenase (5-LO) enzyme, preventing therefore the production of leukotrienes (Berger et al., 2007). The induction of type I IFN production in Mtb-infected mice by treatment with a TLR3 agonist administration results in increased mortality due to uncontrolled bacterial proliferation and immunopathology (Antonelli et al., 2010). The administration of zileuton to Mtb-infected animals treated with poly-ICLC resulted in reduced bacterial loads and lung pathology, consequently promoting improved resistance to Mtb infection in such scenario (Mayer-Barber et al., 2014). These results suggest that in cases of high type I IFN production, such as during viral-Mtb co-infections, inhibition of host 5-LO might be an effective HDT for TB.

Tofacitinib an anti-inflammatory drug that inhibits the activity of Janus kinases located in the cytoplasmic portion of several cytokine receptors, therefore blocking their activation (Winthrop, 2017). BALB/c Mtb-infected mice treated with tofacitinib in conjunction with antibiotics cleared the infection more rapidly than animals that were administered the standard therapy alone. However, the authors did not confirm the involvement of anti-inflammatory effects of tofacitinib in the observed results (Maiga M. et al., 2015).

Inhibitors of Phosphodiesterases (PDE) have also been tested in experimental models and clinical trials as HDTs for TB. The administration of the PDE-4 inhibitor CC-3052 to Mtb-infected mice and rabbits, as well as the PDE-4 inhibitor CC-11050 to Mtb-infected rabbits, in conjunction with isoniazid, resulted in reduced bacterial loads as compared to animals treated with the antibiotic alone and the beneficial effects were associated with reduction in lung pathology and pro-inflammatory responses (Koo et al., 2011; Subbian et al., 2011a; Subbian et al., 2011b; Subbian et al., 2016). The administration of the PDE-4 inhibitor Roflumilast in conjunction with isoniazid also resulted in reduced pulmonary bacterial loads in Mtb-infected mice compared to treatment with INH alone (Maiga M. C. et al., 2015). A clinical trial undertaken in South Africa, cited previously with respect to vitamin D2 supplementation, also identified that the adjunctive treatment of pulmonary TB patients with CC-11050 (PDE-4 inhibitor) added to the standard antibiotics resulted in improved lung function as compared to treatment with antibiotics alone. This study also identified a similar outcome following supplementation with everolimus, an immunosuppressor drug (Wallis et al., 2021). In addition, studies have identified that the treatment of Mtb-infected mice with the selective PDE-3 and 5 inhibitors (cilostazol and sildenafil respectively) added to an antibiotic cocktail containing Rifampicin, Isoniazid and Pyrazinamide, resulted in reduced bacterial loads and lung pathology followed by faster bacterial clearance compared to treatment with antibiotics alone (Maiga et al., 2012; Maiga et al., 2013).

Corticosteroids have also been tested in numerous clinical trials as adjunctive treatments to temper detrimental inflammation in different forms of TB. As reviewed by Schutz et al., the results of trials in which corticosteroid treatment was administered to patients with pulmonary TB vary significantly and it is not possible to draw a solid conclusion regarding possible benefits (Schutz et al., 2018). However, compared to antibiotic therapy alone, the adjunctive treatment with corticosteroids results in decreased mortality rates in tuberculous meningitis (Kumarvelu et al., 1994; Thwaites et al., 2004; Prasad and Singh, 2008; Prasad et al., 2016) and is also beneficial in TB pericarditis (Mayosi et al., 2002; Wiysonge et al., 2017). In both of these conditions, inflammation plays a critical role in disease severity (Wilkinson et al., 2017; Isiguzo et al., 2020), and therefore, anti-inflammatory strategies might represent the best HDTs to be employed.

With the exception of the study by Mayer-Barber et al., which used zileuton to block 5-LO as an HDT for TB and identified an important role for PGE2 and IL-1β in the beneficial effects of the intervention (Mayer-Barber et al., 2014), most of the studies that used anti-inflammatory strategies as HDTs for TB did not characterize the precise mechanisms involved. Therefore, although several HDT candidates that use anti-inflammatory strategies as approaches have been identified, additional studies aimed at identifying the underlying mechanisms are necessary. With that, it will be possible to design fine-tuned new strategies that can be reliably tested and subsequently used in humans.

### Modulation of Granuloma Structure

The formation of granulomas is a hallmark of pulmonary TB and have an important role in the control of bacterial replication and spread (Ulrichs and Kaufmann, 2006). However, as mentioned previously, the granuloma structure can prevent the penetration of antibiotics in the central core (Dartois, 2014) where infected cells reside, as well as impair the access of activated T lymphocytes to these same cells (Egen et al., 2008; Egen et al., 2011). TNF is a key cytokine involved in the formation and maintenance of granulomas during TB and the absence of TNF signaling results in disruption of granulomas and dissemination of bacteria (Flynn et al., 1995; Algood et al., 2005). However, in the context of antibiotic treatment of Mtb-infected mice, the neutralization of TNF administration was shown to enhance the bacterial clearance and decrease immunopathology in comparison to antibiotic treatment alone (Skerry et al., 2012; Bourigault et al., 2013). Also, it was identified that thalidomide and its analogues are potent inhibitors of TNF production (Casal et al., 2016). Accordingly, concomitant administration of thalidomide with antibiotics to TB patients resulted in improved weight gain compared to antibiotic therapy alone (Tramontana et al., 1995). The administration of Etanercept to patients with HIV-TB coinfection undergoing antibiotic treatment also resulted in increases in body mass, decrease in lung involvement and reduced time to sputum conversion and closure of lung cavities (Wallis et al., 2004). The inhibition of TNF is thought act by disrupting TB pulmonary granulomas and consequently enhancing drug penetration but also causing bacterial reactivation, which increases their susceptibility to the action of antibiotics. The administration of thalidomide and its analogs in conjunction with antibiotics was also shown to be beneficial during the treatment of TB meningitis in experimental models (Tsenova et al., 1998; Tsenova et al., 2002) and in patients (van Toorn et al., 2021), although in these cases, the beneficial effects were not associated with granuloma disruption, but likely with the anti-inflammatory effects of thalidomide-induced suppression of TNF production.

The production of vascular endothelial cell growth factor (VEGF), which has angiogenic properties, is enriched in the walls of granulomas surrounding the necrotic cores of humans and rabbits with TB, where abnormal blood vessels are also found. The treatment of Mtb-infected rabbits with bevacizumab, a monoclonal VEGF neutralizing antibody, resulted in normalization of granuloma-associated blood vessels, which promoted increased oxygenation of granulomas and more effective delivery of a molecular tracer into the granuloma necrotic core (Datta et al., 2015). In mice infected with Mtb, neutralization of VEGF or its receptors VEGFR1 and VEGFR2 resulted in decreased extrapulmonary dissemination (Polena et al., 2016), while VEGF genetic deletion in myeloid immune cells resulted in decreased pulmonary inflammation and prolonged survival following Mtb infection, also in mice (Harding et al., 2019). These results suggest that alterations in granuloma structure resulting from neutralization of VEGF or inhibition of VEGF signaling may promote improved drug penetration in granulomas and ameliorate pulmonary tissue damage.

Metalloproteases (MMPs) comprise a family of enzymes that catalyze the degradation of extracellular matrix components, such as collagens, laminin, fibronectin, vitronectin, and proteoglycans (Parks and Shapiro, 2001). In particular, the production of MMP-9 plays an important role in the development of granulomas during Mtb and *M. marinum* infections (Taylor et al., 2006; Volkman et al., 2010). The treatment of Mtb-infected guinea pigs with doxycycline, an antibiotic that acts as a broad spectrum MMP inhibitor, resulted in reduction of pulmonary bacterial loads (Walker et al., 2012). Subsequently it was identified that Mtb-infected mice treated with broad spectrum MMP inhibitors or specific MMP-9 inhibitors adjunctively to isoniazid or rifampicin, displayed decreased pulmonary bacterial loads compared to animals treated with the antibiotics alone. The beneficial effect of MMP inhibition was associated with enhanced vascularization of granulomas and improved delivery and retention of anti-TB drugs in lesion sites (Xu et al., 2018). Additionally, the results of a clinical trial recently published demonstrated that the administration of doxycycline in conjunction with standard antibiotic treatment reduced pulmonary tissue destruction and cavitation in comparison to antibiotic treatment alone, however, no difference in sputum bacterial loads were found (Miow et al., 2021).

The HDT approaches for TB targeting the granuloma structure discussed here benefit in particular the penetration of drugs in lesion sites and optimize their mycobactericidal efficacy, specifically in pulmonary TB. It is tempting therefore to speculate whether the combination of such strategies with others that boost host immunity might link improved access of activated T lymphocytes to granuloma necrotic core with enhanced effector function of these cells, resulting in even more effective control of infection, when performed in conjunction with antibiotics. Moreover, this kind of combinatorial approaches are particularly attractive considering that the strategies that target granuloma structure rely on the effectiveness of antibiotics, since granuloma disruption alone is detrimental for the control of Mtb infection, as mentioned previously (Ulrichs and Kaufmann, 2006).

### Most Promising HDTs for Tuberculosis

Besides the HDT strategies for TB reviewed in this article, several additional approaches have been proposed based on *in vitro* results or in experimental evidences that demonstrated the importance of given biological processes in the modulation of TB pathogenesis. However, further *in vivo* validation is still necessary for many of those, and therefore, such studies were not included.

Regarding the TB HDTs discussed in this review, several have been extensively tested in experimental models and also in clinical trials with positive results, such as those employing metformin, statins, NSAIDs and PDE-4 inhibitors for pulmonary TB as well as corticosteroids for TB-meningitis, and therefore, represent the most promising strategies so far. An important characteristic common to all of these strategies, which also contributes to making them the most promising ones, lies in the fact that they employ drugs have already been approved for human use and can be easily repurposed.

## Host-Directed Therapies for Malaria

The aetiologic agent of malaria, *Plasmodium*, is transmitted by the bite of female *Anopheles* mosquitoes. *P. falciparum* and *P. vivax* are the most common species that cause the disease worldwide, while *P. falciparum* is the most virulent of them, being responsible in some cases for the development of the most severe form of the disease, which manifests as cerebral malaria (CM) (Dondorp et al., 2008; White et al., 2014). *Plasmodium* species have a complex biology that includes liver and blood stages of infection in the mammal host. During blood meal, infected mosquitoes of the *Anopheles* genus inject sporozoite forms of the parasite into the skin, which first gain access to the bloodstream and subsequently to the liver, the primary organ affected by the disease. During the initial steps of the infection, sporozoites stay in the skin for several hours before the beginning of the hepatic stage. Intravital microscopy studies in mice infected with *Plasmodium* species that cause rodent malaria demonstrated that the interaction between sporozoites and several cell types in the skin occur (Hopp et al., 2015) (Vanderberg and Frevert, 2004). As a result, around 50% of the parasites do not leave the inoculation site (Guilbride et al., 2012).

In this early stage of infection, the recently approved RTS, S/AS01 (RTS, S) vaccine could be an interesting strategy to contain disease development, since antibodies found in the skin tissues can inhibit sporozoite motility in the dermis (Sinnis and Zavala, 2012; Renia and Goh, 2016). The RTS, S vaccine targets the circumsporozoite protein (CSP), which is an essential and multifunctional molecule that is linked to the plasma membrane of *Plasmodium* parasites *via* a glycosylphosphatidylinositol (GPI) anchor (Wang et al., 2005). A clinical trial study demonstrated that the use of RTS, S provides partial protection against malaria in young children. So far, this is the only malaria vaccine approved for human use (Rts, 2015). The immobilization of parasites in the skin by the vaccine-induced antibodies against the CSP protein consequently prevents the development of the following phase of infection, which is the liver stage (Vanderberg and Frevert, 2004). To leave skin and enter the blood vessels, sporozoites actively traverse through endothelial cells towards the bloodstream (Mota et al., 2001). Although there is still little information on the precise mechanisms involved, these first phases of infection in which *Plasmodium* parasites need to actively pave their way into the circulation might likely involve interactions with host proteins that are necessary for this process to occur, and, therefore, they also represent the first potential target for host-directed interventions in malaria.

Once in the circulation, the parasites can reach the liver, where they actively transverse endothelial cells towards the organ parenchyma and further invade hepatocytes, developing first into schizonts and later into merozoites (Mota et al., 2001; Hopp et al., 2015). This developmental stage of *Plasmodium* parasites involves several processes that also represent potential targets for HDT interventions in malaria. In *P. falciparum* malaria, the development into schizonts and merozoites starts around the second day after initial liver infection and is completed around the seventh day. However, the duration of hepatic phase of infection can vary among species. During the hepatic stage of malaria, several host pathways have been found to be important for proper parasite development and further completion of its life cycle (Burrows et al., 2017; Lu and Derbyshire, 2020). However, in malaria caused by infection with *P. ovale* and *P. vivax* in particular, there is an additional challenge for possible interventions during the liver stage, because such species can also develop into dormant forms in hepatocytes, the hypnozoites, which can restart the liver cycle weeks, months, or even years after the initial infection occurs, leading to relapse of the disease. Regardless, studies have found that hypnozoites are metabolically active and increase in size slightly over time, and therefore, this form of the parasite can also be targeted therapeutically (Mikolajczak et al., 2015). Until now there are two drugs able to target hypnozoites that are part of the 8-aminoquinoline family. The use of these medicines however is restricted in individuals with some Glucose-6-Phosphate Dehydrogenase (G6PD) polymorphisms (Beutler, 1959; Newby et al., 2015; Baird, 2019; Hounkpatin et al., 2019). Recently, the elucidation of the transcriptomes of *P. vivax* and *P. cynomolgi* (a parasite species that causes malaria in nonhuman primates) contributed to a better understanding of the host processes and metabolites that are critical for parasite development (Voorberg-van der Wel et al., 2017; Gural et al., 2018). That way, additional studies on such interactions can highlight host pathways that are critical for the maintenance of hypnozoites, and, therefore, guide the development of novel host-directed therapeutic strategies that can target the pathogens in this stage of their life cycle.

After the development within the liver is complete, merozoites are released into the bloodstream as merosomes. During this process, vesicles containing parasites form and bud off from the infected hepatocytes, and, once the merosomes are released from the vesicles into the bloodstream, they can actively invade red blood cells (RBCs) (Kappe et al., 2003; Heussler et al., 2006; Ashley et al., 2018). Inside erythrocytes, *Plasmodium* parasites modify the cell membrane by exporting a series of proteins and reorganizing the host cell with various structures including Maurer’s clefts, Schüffner’s dots, and knobs, all of which facilitate the adhesion of infected erythrocytes to endothelia, particularly in central nervous system capillaries, an effect that is intimately involved in the induction of inflammatory response-induced cerebral malaria pathogenesis (Kilejian, 1979; Mundwiler-Pachlatko and Beck, 2013). Inside RBCs, merozoites differentiate into trophozoites, which replicate and form schizonts. RBCs then burst and release new merozoite forms in the bloodstream that can infect new uninfected erythrocytes. Some of these merozoites develop into sexual forms (male or female gametocytes) that can be transmitted to a mosquito during a blood meal (Ashley et al., 2018). The RBC rupture event is what triggers the episodic fever, a hallmark of malaria, which is caused by the high production of pro-inflammatory cytokines by the host in response to the systemic release of agonists of innate immune cell receptors (Parroche et al., 2007).

Therefore, while first line host-targeted interventions could prevent sporozoites from invading hepatocytes and RBCs, a second line strategy could be aimed at arresting parasite development inside the host cells, which could also promote protective immunity, as it has been observed during infection with live attenuated *Plasmodium* strains (Leiriao et al., 2005). A third line of intervention for HDTs against malaria can focus on the modulation of host-detrimental inflammatory responses, in particular those developed during *P. falciparum* infection-induced cerebral malaria.

**Figure 2:**
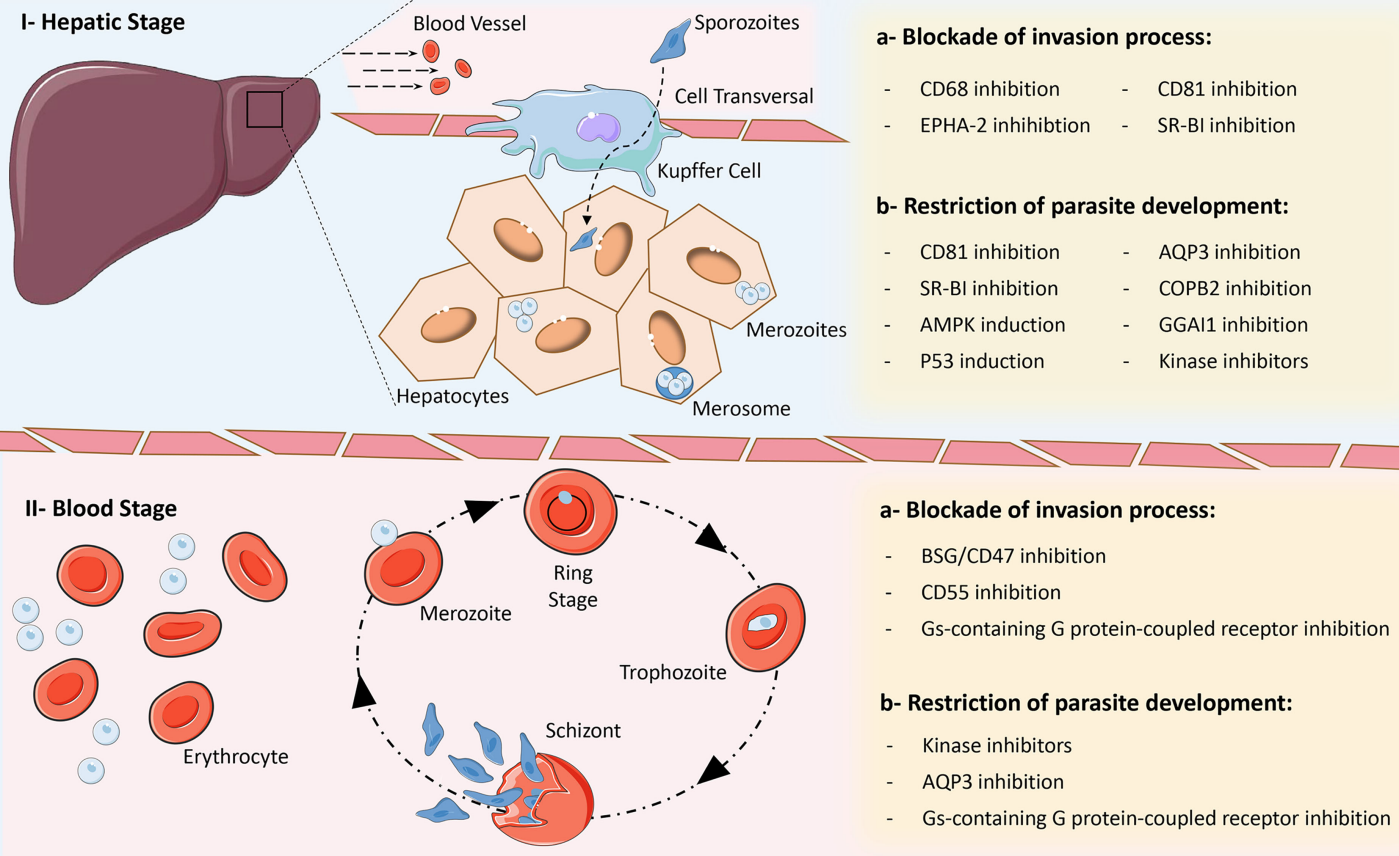
Main strategies for the development of Host-Directed Therapies for malaria. Due to complex biology of *Plasmodium* parasites, several opportunities for intervention within biological processes exist in the different phases of the parasite’s life cycle, which can block cell invasion and promote improved parasite clearance or restrict their development within host cells. I – Hepatic stage: a) inhibition of CD68, EPHA-2, CD81 and SR-BI can block the invasion of hepatocytes by different *Plasmodium* species; b) inhibition of CD81, SR-BI, AQP3, COPB2, GGAI1 and the activity of some kinases, as well as induction of p53 and AMPK expression, can restrict the development of different *Plasmodium* species within hepatocytes. II – Blood stage: a) inhibition of BSG/CD47, CD55 and activity of Gαs-containing G protein-coupled receptors can block the invasion of red blood cells by different *Plasmodium* species; b) inhibition of the activity of some kinases and Gαs-containing G protein-coupled receptors, as well as blockade of the AQP3 receptor can restrict the development of different *Plasmodium* species within red blood cells. Some elements in this figure use pictures from Servier Medical Art (https://smart.servier.com) licensed under a Creative Commons Attribution 3.0 Unported License (https://creativecommons.org/licenses/by/3.0/).

In the next section, we discuss the processes in different stages of infection with *Plasmodium* species that involve the participation of host molecules and can therefore represent potential targets for the development of new HDTs to combat malaria (**Figure 2**).

### Blockade of Host Cell Receptors Used for Parasite Invasion

Interactions between sporozoites and the host receptors are potential targets for interventions due to their important role in parasite development. After inoculation into the skin, sporozoites must break several physical barriers to advance in their life cycle. The first of those is done through the process of cell transversal, which allows sporozoites to cross host cell membranes and other cellular barriers (Tavares et al., 2013). As mentioned previously, after being carried to the liver in the blood vessels, sporozoites again need to traverse the sinusoidal endothelium, a process in which Kupffer cells also participate, for further infection of hepatocytes (Frevert et al., 2006; Amino et al., 2008). CD68 is a transmembrane glycoprotein expressed in the endosomes and lysosomes of cells from the monocyte lineage. Glyceraldehyde 3-Phosphate Dehydrogenase (GAPDH) expressed on the surface of sporozoites bind to CD68 expressed by liver resident Kupffer cells, which facilitates the transversal process of *Plasmodium* parasites towards the liver (Cha et al., 2016). Importantly, it was found that blockade of CD68 substantially impairs liver infection by *P. berghei* parasites, therefore demonstrating that host CD68 represents an interesting target for host-based intervention (Cha et al., 2015).

HDTs could also be implemented in order to prevent the infection of hepatocytes or erythrocytes by the parasites trough targeting host cell receptors that are directly required for the pathogen to invade these cells. Targeting hepatocytes is particularly attractive, since this will also prevent the further infection of erythrocytes and subsequent transmission. However, defining target candidates for intervention in this step of infection is very challenging due to the vast heterogeneity regarding susceptibility to *Plasmodium* infection observed in hepatocytes from distinct mouse strains as well as from different human donors (March et al., 2013; Kaushansky et al., 2015a; Roth et al., 2018). Additionally, it was observed that the suceptibility of hepatocytes to *Plasmodium* infection can vary even within a single individual (Austin et al., 2014). An additional challenge lies in the fact that distinct species of the parasite, such as *P. falciparum* and *P. vivax* use different receptors for hepatocyte invasion (Manzoni et al., 2017). Regardless, multiple host receptors and several cell intrinsic properties have been demonstrated to be required for the multistep complex process of *Plasmodium* entry into hepatocytes, which can be targeted by host-directed interventions.

CD81 is a transmembrane glycoprotein expressed on the surface of hepatocytes that is part of the tetraspanin receptors family, which was described to be required for both human *P. falciparum* and rodent *P. yoelii* sporozoite entry into hepatocytes and further formation of the parasitophorous vacuole (Silvie et al., 2003). The scavenger Receptor BI (SR-BI) is a membrane protein that mediates selective cellular uptake of cholesterol and is expressed in hepatocytes, Kupffer cells, and hepatic sinusoidal endothelial cells (Acton et al., 1996; Rhainds and Brissette, 2004), which was also described to have a role both in *P. berghei* (which induces experimental severe malaria in rodents) and *P. falciparum* sporozoite invasion and development within hepatocytes (Rodrigues et al., 2008; Yalaoui et al., 2008). It was later identified that *Plasmodium* parasites use members of the *Plasmodium* 6-cysteine domain protein family to interact with CD81 or SR-BI and invade liver cells. Genetic deletion of P36 and P52, which are members of the protein family mentioned above, restricts hepatocyte invasion through both CD81 and SR-BI by *P. berghei* and *P. yoelli*, while deletion of P36 alone abrogates *P. berghei* entry into hepatocytes *via* SR-BI (Manzoni et al., 2017). However, the characterization of CD81 crystal structure revealed that this molecule can also bind cholesterol molecules (Zimmerman et al., 2016) and mice genetically deficient for SR-BI display decreased expression of CD81 (Yalaoui et al., 2008). Therefore, it is possible that both CD81 and SR-BI may promote parasite development by not only favoring the invasion of cells, but also by providing cholesterol to the parasitophorous vacuole, and therefore, inhibition of this pathway can be used as HDT strategy to both inhibit invasion or starve the parasite from cholesterol. Kaushansky et al. found that *P. yoelli* parasites also use P36 to interact with the receptor Ephrin type-A receptor 2 (EPHA-2) in hepatocytes and invade these cells. In addition, the authors found that EphA2-deficient mice are less susceptible to infection with *P. yoelii* sporozoites (Kaushansky et al., 2015b), indicating that EPHA2 represents a potential target for HDT to block the entry of parasites in hepatocytes.

As mentioned previously, during the following phase of *Plasmodium* infection, merozoites released from the liver enter the bloodstream and begin the blood stage of the disease, which includes erythrocyte invasion, intraerythrocytic growth, replication and egress from infected RBCs. *P. falciparum* infects mature erythrocytes, while *P. vivax* preferentially infects reticulocytes. The current drugs used in malaria treatment target mainly these forms of the parasite that replicate asexually in the blood and are responsible for most of the characteristic symptoms of malaria. The development of host-directed strategies to block erythrocyte invasion can therefore potentially augment the elimination of blood stage *Plasmodium* achieved by parasite-targeted drugs. In addition, pathogens resistant to the antimalarials, which represent a major challenge for malaria treatment, will continue to be equally susceptible to HDTs.

In the 1970’s it was described that the Duffy receptor expressed in erythrocytes was implicated in the susceptibility to infection with *P. vivax* in individuals of African descent (Miller et al., 1976), which could place this antigen as a potential target for host-targeted therapeutical interventions. However, although the Duffy antigen is indeed used for the invasion of erythrocytes by the parasite, in the current days, multiple cases of *P. vivax* infection have been reported in Duffy-negative populations (Gunalan et al., 2018). Therefore, the identification of more reliable targets is needed. In this sense, basigin (BSG or CD47) is used for erythrocyte invasion following the binding to *P. falciparum* reticulocyte binding homolog 5 (PfRh5), expressed on the pathogen’s surface. The knocking down of basigin in erythrocyte progenitors, as well as the addition of recombinant basigin or anti-basigin antibodies to human erythrocyte cultures, substantially restricted *P. falciparum* invasion of RBCs *in vitro* (Crosnier et al., 2011). CD55 (complement decay-accelerating factor - DAF) was also demonstrated to be required for the invasion of erythrocytes by *P. falciparum* merozoites. Knocking down of CD55 in RBC progenitors resulted in significant inhibition of *P. falciparum* invasion of the mature erythrocytes in further *in vitro* experiments (Egan et al., 2015). The precise mechanisms through which CD55 promotes RBC invasion are not completely characterized. Nonetheless, a recent study found that CD55 is engaged after *P. falciparum* discharges the contents of rhoptries towards erythrocytes and that the receptor may play a role in the formation of the moving junction between parasite and erythrocyte membranes (Shakya et al., 2021). These studies therefore suggest that basigin and CD55 from erythrocytes can be could be used as targets for HDTs in malaria to block the invasion of RBCs by the parasites. In addition, the signaling through Gαs-containing G protein receptors from erythrocytes was shown to play an important role during merozoite invasion of these cells and also for the process of intracellular growth of the blood stage parasites. Moreover, in this study, the authors demonstrated that treatment of infected erythrocytes with propranolol and other β-blockers (all of which inhibit the signaling of β adrenergic G protein-coupled receptors) inhibit intraerythrocytic parasite growth (Murphy et al., 2006). The precise mechanisms involved in this effect are not fully described, however, the results of this study indicate that inhibitors of Gαs-containing G protein receptors can be repurposed for use as HDTs that specifically block the erythrocytic phase of *Plasmodium* infection.

The studies cited above clearly highlight the complex interplay that occurs between pathogen and host molecules, which are required for successful *Plasmodium* infection. Such mammal host proteins that are used as receptors or that favor the process of parasite invasion of cells can be targeted for the development of HDTs that neutralize pathogen entry and therefore have the capacity to block *Plasmodium* infection, as well as malaria development and transmission.

### Inhibition of Parasite Growth in Host Cells

Once sporozoites invade hepatocytes, certain cellular biological processes from the host are critical for the intracellular development of the liver stage parasites. There are many drugs that act as inhibitors of such cellular processes and are commonly used for the treatment of other conditions. Those drugs can therefore be easily repurposed and used to target the complex parasite growth process, serving as novel HDTs for the treatment of malaria.

Metabolic components from the host cell are necessary for the proper development of *Plasmodium* parasites. A better understanding of the alterations in host cell metabolic state caused by the infection can reveal novel targets for HDT strategies that control the development of malaria. *Plasmodium* parasites use the fatty acid synthesis machinery of host cells in order to grow in the liver (Mikolajczak et al., 2007; Itoe et al., 2014). Considering that, the tumor suppressor protein p53 can be considered a potential host target, since it was described to play an important role in the control of lipid peroxidation process in hepatocytes (Kain et al., 2020). In fact, the increased levels of p53 expression were shown to result in improved control of parasite replication in the liver (Kaushansky et al., 2013b; Douglass et al., 2015).

It was demonstrated that the induction of endoplasmic reticulum (ER) stress in hepatocytes favors *Plasmodium* infection in the liver (Inacio et al., 2015; Kaushansky and Kappe, 2015). The induction of AMP-activated protein kinase (AMPK) activity can prevent accumulation of lipids as well as ER stress in hepatocytes (Li et al., 2014), promoting improved control of *Plasmodium* liver infection (Ruivo et al., 2016), and therefore, AMPK induction strategies may represent additional potential HDTs that can restrict parasite growth in liver cells. The coatomer protein complex I (COPB2, COPG1) is responsible for the retrograde vesicular trafficking from the Golgi to ER, while the ADP-ribosylation factor-binding protein (GGA1) in involved in the trafficking of proteins from the trans-Golgi network to endosomes (Paczkowski et al., 2015). A study identified that the disruption of intracellular trafficking mediated by these modulators restrains parasite development in hepatocytes, likely by restricting the access of parasites to important host-derived molecules in the parasitophorous vacuole. Therefore, such intracellular transporters represent potential targets for intervention in the treatment of malaria (Raphemot et al., 2019). Another possible target for HDT development in malaria, which is involved in the transport of water, glycerol and H_2_O_2_ through cell membranes, is the transmembrane channel aquaporin-3 (AQP3). Studies have identified that the recruitment of this transporter to the parasitophorous vacuole occurs in hepatocytes and erythrocytes infected with *Plasmodium* parasites and that inhibition of AQP3 reduces parasite burdens in liver as well as in the blood (Posfai et al., 2018; Posfai et al., 2020).

Kinases represent a substantial proportion of key essential intracellular components that govern all kinds cellular metabolic processes. It was identified that during malaria, 47 host kinases play important roles in promoting parasite growth (Arang et al., 2017). Several kinase inhibitors have been approved for human use, primarily for the treatment of cancer and autoimmune disorders (Ferguson and Gray, 2018). Some host kinases are involved in the development of both hepatic and blood stages parasites. The phospho-signaling pathway, which involves the host cell kinases PAK (p21-activated kinase) and MEK1 (MAP/ERK kinase) was shown to be activated during infection with *P. falciparum*. The authors further identified that the treatment with U0126, a MEK inhibitor, impaired parasite proliferation in both hepatocytes and erythrocytes (Sicard et al., 2011). Others kinases that are known to be activated during *Plasmodium* infection and that can be targeted by inhibitors as potential HDTs to restrain intracellular parasite replication include the TGF-β receptor 1 (Arang et al., 2017), Src family kinases (Gillrie et al., 2007; Kaushansky et al., 2013a), Rho-associated protein kinase (Taoufiq et al., 2008), Pak1 and MEK1/2 (Sicard et al., 2011; Kesely et al., 2016), B-Raf and c-MET/Alk (Adderley et al., 2020).

### Modulation of Immune and Inflammatory Responses

After hepatocyte invasion, *Plasmodium* parasites replicate exponentially until the release of merozoites. During this process, activation of several immune effector mechanisms occurs, such as complement fixation, phagocytosis or lysis of infected cells by cytotoxic NK and NKT cells. The recognition of parasite neoantigens at the surface of infected hepatocytes by antibodies also mediates the killing of such cells through an antibody-dependent cell-mediated mechanism by Kupffer cells and NK cells (Casares and Richie, 2009). In fact, the pathogenesis of malaria and symptomatology of the disease is intimately correlated with the production of elevated levels of proinflammatory cytokines by the infected host, such as TNF, IL-1, IFN-γ, as well as chemokines, such as CXCL10 and CXCL4, which are particularly important in cerebral malaria, which is the most severe manifestation of the disease (Grau et al., 1989; Kwiatkowski et al., 1990; Wilson et al., 2011). In this sense, immunomodulatory host targeted therapy seems to be a logical approach, especially concerning the management of the most severe manifestations of *Plasmodium* infection in cerebral malaria.

Rosiglitazone is an agonist of the peroxisome- activated receptor γ (PPAR-γ), which promotes reduction of inflammatory responses. Its use in patients with uncomplicated *P. falciparum* malaria resulted in accelerated parasite clearance from blood as well as lower production of pro-inflammatory mediators namely IL-6 and MCP-1, in comparison to patients who were administered placebo (Boggild et al., 2009). Due to these beneficial effects, a clinical trial was developed to assess the efficacy of rosiglitazone treatment in patients with severe malaria, for which the results were not yet published (NCT02694874).

Using an experimental model of infection with *P. berghei* ANKA, Pamplona et al., identified that the induction of HO-1 expression through the treatment with CoPPIX as well as the administration of CO, which is an end product of HO-1-mediated heme degradation, protects mice against severe lethal cerebral malaria, (Pamplona et al., 2007). HO-1 is widely known for its anti-inflammatory and antioxidant properties. The nuclear factor erythroid 2–related factor 2 (NRF2) pathway is a major inducer of HO-1 expression along with several other genes encoding antioxidant proteins (Costa et al., 2020), and the administration of an inducer of this pathway is being used in the treatment of multiple sclerosis and psoriasis (Gopal et al., 2017). Therefore, the NRF2-HO-1 pathway along with its anti-inflammatory and antioxidant properties represent a potential target for the development of HDTs focused on the modulation of inflammatory responses that are implicated in the endothelial dysfunction and tissue damage observed in cerebral malaria.

However, although the use of anti-inflammatory strategies has yielded promising results in experimental models and in uncomplicated malaria, clinical trial with several immunomodulatory approaches have been largely unsuccessful in cerebral malaria, as reviewed elsewhere (Varo et al., 2018). These data therefore indicate that the modulation of inflammatory and immune responses in the different manifestations of malaria caused by the distinct species of *Plasmodium* require further characterization before novel anti-inflammatory interventions can be tested in clinical trials.

### Most Promising HDTs for Malaria

Differently from TB, not many HDT strategies have been tested *in vivo* for malaria and the majority of the proposed therapeutic approaches is based on *in vitro* models using parasites that infect humans or in evidences gathered from experimental models in which rodent *Plasmodium* species were used. Nonetheless, some approaches seem more feasible to be employed and hold promise to be more effective. In particular, strategies aimed at blocking the invasion and development of parasites in erythrocytes are mostly attractive, since that is the developmental stage of the parasites in the host in which disease symptoms develop and also when transmission to the invertebrate vector occurs (White et al., 2014). Importantly, some of the cited strategies that target this phase employ drugs that are already approved for use in humans and can be easily repurposed, such as propranolol and β-blockers as well as different kinase inhibitors.

## Tuberculosis and Malaria Co-infection

There are several countries, especially in sub-Sharan Africa, in which the incidence of both TB and malaria are high (WHO, 2019; WHO, 2021). As an example, a retrospective study published by Valadas et al. found that 37.4% of the TB patients admitted in a hospital in Luanda, the capital of Angola, were co-infected with *Plasmodium* species, most of them with *P. falciparum* (Valadas et al., 2013). A study by Colombatti et al. found that malaria co-infection increases the mortality rates of severely ill TB patients (Colombatti et al., 2011), however, there are not many published studies that specifically investigated TB-malaria co-infection pathogenesis in humans.

Using murine models of infection, the role of immunomodulation in the pathogenesis of both TB and malaria has been investigated in some studies. In accordance with the data obtained from humans cited above, Scott et al. found that co-infection of mice with *M. tuberculosis* and *P. yoelii* resulted in impaired control of Mtb replication and higher mortality due to experimental TB (Scott et al., 2004). Using different *Plasmodium* species, Hawkes et al. found that Mtb-infected mouse macrophages display impaired control of bacterial replication following incubation with *P. falciparum*-infected erythrocytes. In addition, the co-infection of Mtb-infected C57BL/6 mice with *P. chabaudi* resulted in higher spleen and liver bacterial loads, which was associated with the accumulation of hemozoin in granuloma phagocytes, rendering these cells less effective in controlling Mtb replication (Hawkes et al., 2010). The co-infection with *P. berghei* in Mtb-infected mice was also found to exacerbate experimental TB, resulting in higher bacterial loads, inflammatory and immune responses. On the other hand, co-infected mice displayed more effective control of *Plasmodium* replication (Mueller et al., 2012), suggesting that the enhancement in immune response magnitude promoted by Mtb favors the control of experimental malaria. In fact, the infection with *P.yoelli* in C57BL/6 mice previously infected with Mtb resulted in more effective control in parasitemia and increased survival to experimental malaria, which was associated with enhancement of Th1 immune responses (Page et al., 2005). Accordingly, BCG (attenuated *M. bovis*) vaccination was found to induce protection of A/J mice from lethal *P. yoelii* infection (Matsumoto et al., 2000). BCG vaccination also protected A/J mice from *P. chabaudi* infection, however, in C57BL/6 mice, BCG vaccination resulted in higher susceptibility and mortality following *P. chabaudi* infection (Leisewitz et al., 2008).

Considering the studies cited above, it is evident that the immunomodulation resulting from either *Plasmodium* or Mtb infections can impact the pathogenesis of both TB and malaria in co-infection scenarios. Particularly in malaria, the results obtained from murine models, although still debatable, suggest that the immunostimulatory properties of Mtb and other mycobacteria can promote improved control of parasite replication, indicating that HDT strategies based on therapeutic vaccination with mycobacterial cells or products may hold promise to improve host resistance to malaria. This approach however, needs to be carefully addressed, especially in the cases of severe malaria, in which excessive inflammation is detrimental (White et al., 2014). On the other hand, the cited studies indicate that *Plasmodium* co-infection impairs host control of bacterial replication, supposedly by dampening immune responses. Nonetheless, further studies aimed at addressing the mechanisms involved, may identify *Plasmodium*-derived products that may be useful as HDTs for TB that are focused on restraining inflammation-driven tissue damage.

## Concluding Remarks

Although tuberculosis and malaria differ in several aspects, together, these diseases have accounted for the majority of deaths caused by infectious agents in the last centuries (Paulson, 2013). Populations that live in conditions of vulnerability, such as lack of access to proper nutrition, sanitation and basic health care, are the ones at higher risk of acquiring these diseases. In fact, high incidences of both malaria and TB occur in several low-income countries (de Araujo et al., 2020), in which cases of coinfection with both *Plasmodium* and *M. tuberculosis* must be common, although, probably, sub notification of such cases must occur.

In the recent years, the knowledge regarding host-pathogen interactions in both of these diseases have been considerably expanded and revealed several host biological processes that favor the progression of infection and disease development. Such processes can therefore be targeted with immunopharmacological interventions for the development of host-directed therapies to treat these diseases. In this article we have reviewed such potential targets and strategies in TB and malaria, which can also be accessed in **Tables 1**, **2**, respectively. These approaches present the advantage of being also active against drug resistant pathogens, because they target the host, not the microorganism. However, due to this same reason, the possibility of development of toxicity and more severe side effects is also a concern for new proposed HDTs. On the other hand, several drugs approved for use in the treatment of other illnesses can be repurposed for HDTs in several of the cases discussed in the present review, which can save time, effort and money, since the safety profile of these compounds has already been extensively characterized. Finally, the HDT approach can expand the available treatment options and considerably improve the efficacy of existing therapeutic regimens, providing therefore an important help in the fight against both TB and malaria.

**Table 1:** Host-Directed therapies for Tuberculosis.

HDT	Outcome	Host	Reference
C40.T4 (TLR4 and CD40 agonist)	Reduction in bacterial loads in conjunction with antibiotics; increased production of IL-12, TNF, IL-6, IFN-γ and IL-17.	Mouse	(Khan et al., 2016)
IL-12	Restoration of responsiveness to antibiotic treatment in disseminated TB.	Human	(Greinert et al., 2001)
IFN-γ and IFN-γ1b	Enhanced rates of negative sputum conversion in conjunction with antibiotics; reduction of inflammation and enhancement of CD4^+^ T cell responsiveness in drug susceptible TB and MDR TB.	Human	(Condos et al., 1997) (Dawson et al., 2009)
IL-2	Enhanced rates of negative sputum conversion in conjunction with antibiotics; enhanced cellular immunity; improved radiological scores in MDR TB.	Human	(Johnson et al., 1997) (Shen et al., 2015) (Tan et al., 2017)
GM-CSF GM-CSF + IL-2	Reduction in bacterial loads in mice and trend to faster negative sputum conversion in humans in adjunction with antibiotics.	Mouse Human	(Zhang et al., 2012) (Pedral-Sampaio et al., 2003)
Metformin	Reduced bacterial loads and tissue inflammation in conjunction with antibiotics; enhanced intracellular bacterial killing, ROS production in Mtb-infected cells and IFN-γ production by CD8^+^ T cells in mice. Reduced bacterial loads in guinea pigs. Reduction of mortality and enhanced rates of negative sputum conversion in humans in conjunction with antibiotics.	Mouse Guinea pig Human	(Singhal et al., 2014) (Lee et al., 2018) (Frenkel et al., 2020) (Yu et al., 2019) (Zhang and He, 2020)
Statins	Reduced bacterial loads; accelerated antibiotic clearance; enhanced autophagy and phagosomal maturation in mice. Reduced risk of TB development in humans.	Mouse Human	(Parihar et al., 2014) (Skerry et al., 2014) (Dutta et al., 2016) (Dutta et al., 2020) (Lee et al., 2015) (Lai et al., 2016) (Su et al., 2017) (Li et al., 2020)
GPR109A inhibition	Reduced lipid body formation, reduced bacterial loads; enhanced microbicidal activity in infected cells.	Mouse	(Singh et al., 2012)
Carbamazepine	Reduced bacterial loads and tissue pathology; enhanced autophagy, TNF, IL-12 and IL-27 production.	Mouse	(Schiebler et al., 2015)
Ibrutinib	Reduced bacterial loads; enhanced autophagy	Mouse	(Hu et al., 2020)
Gefitinib	Reduced bacterial loads; enhanced autophagy and lysosomal biogenesis; reduced STAT3 signaling.	Mouse	(Stanley et al., 2014) (Sogi et al., 2017)
STAT3 and IL-10 inhibition	Reduction of bacterial loads; enhancement of apoptosis and autophagy; increased iNOS and NADPH oxidase activity.	Mouse	(Upadhyay et al., 2018)
Sirtuin 1 activation	Reduced bacterial loads and tissue pathology in conjunction with antibiotics; enhanced autophagy.	Mouse	(Cheng et al., 2017)
Sirtuin 2 inhibition	Reduced bacterial loads in conjunction with antibiotics; enhanced innate and T cell-mediated immunity.	Mouse	(Bhaskar et al., 2020)
HO-1 inhibition	Reduced bacterial loads and accelerated bacterial clearance by antibiotics; enhanced iNOS expression and NO production in response to IFN-γ.	Mouse	(Costa et al., 2016) (Costa et al., 2020b)
Vitamin D supplementation	Induction of cathelicidin-mediated autophagy in human macrophages; enhanced rates of negative sputum conversion in humans in conjunction with antibiotics. Reduced bacterial loads in conjunction with antibiotics in mice.	Human Mouse	(Liu et al., 2006) (Liu et al., 2007) (Yuk et al., 2009) (Wu et al., 2018) (Jolliffe et al., 2019) (Karbalaei et al., 2020)
Retinoic acid supplementation	Reduced bacterial loads and tissue lesions; enhanced recruitment of CD4^+^ and CD8^+^ T cells as well as macrophages to lesions.	Mouse Rat	(Yamada et al., 2007) (O'Connor et al., 2019)
Lactate dehydrogenase inhibition	Reduced bacterial loads in conjunction with antibiotics in iNOS^-/-^ mice.	Mouse	(Krishnamoorthy et al., 2020)
IDO inhibition	Reduced bacterial loads; enhanced T cell immunity.	Non-human primate	(Gautam et al., 2018)
IL-1β signaling inhibition	Reduced tissue inflammation in conjunction with antibiotics.	Mouse Non-human primate	(Mishra et al., 2013) (Winchell et al., 2020)
NSAIDs (aspirin and ibuprofen)	Reduced tissue inflammation and bacterial loads in mice. Reduced mortality in patients with TB-meningitis.	Mouse Human	(Kroesen et al., 2018) (Vilaplana et al., 2013) (Byrne et al., 2007) (Mai et al., 2018)
Zileuton	Reduced bacterial loads and tissue pathology in mice treated with poly-ICLC.	Mouse	(Mayer-Barber et al., 2014)
Tofacitinib	Reduced bacterial loads in conjunction with antibiotics.	Mouse	(Maiga M. et al., 2015)
PDE inhibitors (4, 3 and 5)	Reduced bacterial load and tissue pathology in mice and rabbits in conjunction with antibiotics. Improved lung function in patients in conjunction with antibiotics.	Mouse Rabbit Human	(Koo et al., 2011) (Subbian et al., 2011a) (Subbian et al., 2011b) (Subbian et al., 2016) (Maiga M. C. et al., 2015) (Wallis et al., 2021) (Maiga et al., 2012) (Maiga et al., 2013)
Corticosteroids	Reduced mortality rates in TB-meningitis; beneficial outcome in TB-pericarditis, both in conjunction with antibiotics.	Human	(Mayosi et al., 2002) (Wiysonge et al., 2017)
TNF blockade (immunobiological or thalidomide)	Reduced bacterial load and tissue pathology in conjunction with antibiotics in mice. Improved weight gain and reduced time to negative sputum conversion in conjunction with antibiotics in humans.	Mouse Human	(Skerry et al., 2012) (Bourigault et al., 2013) (Tramontana et al., 1995) (Wallis et al., 2004)
VEGF and VEGFR blockade	Reduced bacterial loads; decreased extrapulmonary dissemination; reduced tissue inflammation.	Mouse	(Polena et al., 2016) (Harding et al., 2019)
Metalloprotease inhibitors (broad spectrum or MMP9)	Reduction of bacterial loads in guinea pigs. Reduction of bacterial loads in conjunction with antibiotics in mice. Reduced tissue pathology in conjunction with antibiotics in humans.	Guinea pigs Mouse Human	(Walker et al., 2012) (Xu et al., 2018) (Miow et al., 2021)

**Table 2:** Host-directed therapies for malaria.

HDT	Outcome	Host	Reference
CD68 blockade	Inhibition of *P. berghei* transversal through Kupfer cells and subsequent liver infection.	Mouse	(Cha et al., 2015)
CD81 blockade	Inhibition of *P. falciparum, P. yoelii and P. berghei* infection and development in liver cells.	Mouse liver cell line Human liver cell line	(Silvie et al., 2003) (Manzoni et al., 2017)
SR-BI blockade	Inhibition of *P. falciparum, and P. berghei* infection and development in liver cells.	Mouse liver cell line Human liver cell line	(Rodrigues et al., 2008) (Yalaoui et al., 2008)
Basigin (CD47) blockade	Inhibition of *P. falciparum* infection in erythrocytes.	Human erythrocytes	(Crosnier et al., 2011)
CD55 blockade	Inhibition of *P. falciparum* infection in erythrocytes.	Human erythrocytes	(Egan et al., 2015) (Shakya et al., 2021)
Propanolol and β-blockers	Inhibition of *P. falciparum* infection and development in erythrocytes.	Human erythrocytes	(Murphy et al., 2006)
p53 expression induction	Restriction of *P. yoelii* and *P. falciparum* growth in liver.	Mouse Humanized mouse	(Kaushansky et al., 2013b) (Douglass et al., 2015)
AMPK induction	Restriction of *P. berghei*, *P. yoelii* and *P. falciparum* growth in liver cells.	Mouse Mouse liver cell line Human liver cell line	(Ruivo et al., 2016)
COPB2 and GGA1 inhibition	Restriction of *P. berghei* and *P. yoelii* growth in liver cells.	Mouse liver cell line Human liver cell line	(Raphemot et al., 2019)
AQP3 inhibition	Restriction of *P. berghei*, *P. falciparum* and *P. vivax* development in liver cells.	Human liver cell line	(Posfai et al., 2018) (Posfai et al., 2020)
MEK (protein kinase) inhibitor	Restriction of *P. berghei* and *P. falciparum* development in liver cells and erythrocytes.	Human liver cell line Human erythrocytes	(Sicard et al., 2011)
Rosiglitazone (PPAR-γ agonist)	Reduced parasitemia in humans infected with *P. falciparum*.	Humans	(Kapadia et al., 2008) (Boggild et al., 2009)
HO-1 induction	Enhanced protection against cerebral malaria in *P. berghei* ANKA-infected mice.	Mouse	(Pamplona et al., 2007)

## Author Contributions

Conceptualization: KM, AC and DC. Figures: KM. Writing: original draft – KM, AC and DC; review and editing: KM and DC. All authors contributed to the article and approved the submitted version.

## Funding

KM is funded by Fundação de Amparo à Pesquisa do Estado de São Paulo (FAPESP) fellowship number 2020/01043-9; AC is funded by Fundação de Amparo à Pesquisa do Estado de São Paulo (FAPESP) fellowship number 2020/10356-0; DC is funded by Fundação de Amparo à Pesquisa do Estado de São Paulo (FAPESP) grant number 2019/08445-8 and fellowship number 2019/25770-0.

## Conflict of Interest

The authors declare that the research was conducted in the absence of any commercial or financial relationships that could be construed as a potential conflict of interest.

## Publisher’s Note

All claims expressed in this article are solely those of the authors and do not necessarily represent those of their affiliated organizations, or those of the publisher, the editors and the reviewers. Any product that may be evaluated in this article, or claim that may be made by its manufacturer, is not guaranteed or endorsed by the publisher.
